# Customizable document control solution for 3D printing at the point-of-care

**DOI:** 10.1186/s41205-023-00172-0

**Published:** 2023-03-17

**Authors:** Maxwell Lohss, Elliott Hammersley, Anish Ghodadra

**Affiliations:** 1 UPMC Department of Radiology, 200 Lothrop St, Pittsburgh, PA 15213 USA; 2 University of Pittsburgh School of Medicine, 3550 Terrace St, Pittsburgh, PA 15213 USA

**Keywords:** 3D printing, Additive manufacturing, Quality management system, Quality control, Document control, Regulatory, CFR Part 11

## Abstract

**Background:**

The rapid expansion and anticipated U.S Food and Drug Administration regulation of 3D printing at the point-of-care necessitates the creation of robust quality management systems. A critical component of any quality management system is a document control system for the organization, tracking, signature collection, and distribution of manufacturing documentation. While off-the-shelf solutions for document control exist, external programs are costly and come with network security concerns. Here, we present our internally developed, cost-effective solution for an electronic document control system for 3D printing at the point-of-care.

**Methods:**

We created a hybrid document control system by linking two commercially available platforms, Microsoft SharePoint and Adobe Sign, using a customized document approval workflow.

**Results:**

Our platform meets all Code of Federal Regulations Title 21, Part 11 guidances.

**Conclusion:**

Our hybrid solution for document control provides an affordable system for users to sort, manage, store, edit, and sign documents. The system can serve as a framework for other 3D printing programs to prepare for future U.S Food and Drug Administration regulation, improve the efficiency of 3D printing at the point-of-care, and enhance the quality of work produced by their respective program.

## Introduction

Point-of-care 3D printing is a growing sector of healthcare, currently being practiced across various academic medical centers and private clinics [1]. Healthcare systems have adopted 3D printing technologies to create patient-specific anatomic models, surgical guides, and other custom medical devices [2]. The use of 3D printing in the clinical environment is improving diagnostic testing, creating new opportunities for surgical planning, decreasing time spent in the operating room, reducing medical errors, and ultimately improving patient care and satisfaction [3–5].

As 3D printing expands into more clinical settings and broadens in its applications, the demand for robust quality control grows. Most 3D printing programs can be divided into three categories. Programs are either (1) developed in collaboration with a separate manufacturing company, (2) established internally under a hospital department, or (3) developed as combination of both external and internal manufacturing facilities [6]. These different implementation strategies, as well as advancements in 3D printing technology, have made 3D printing more accessible for both large and small institutions. To ensure that this rapid growth of 3D printing in healthcare settings continues to be safe and effective, robust quality management infrastructure is critical [7–10]. With the U.S. Food and Drug Administration (FDA) releasing a discussion paper on regulation of 3D printing in 2021, it is only a matter of time until formal regulation is placed on 3D printing programs. As hinted by the FDA, this regulation could be as stringent as that of a traditional medical device manufacturer [6]. The discussion paper also states that 3D printing entities should understand the existing requirements for medical device regulation and comply to the Quality System Regulation under Title 21 Code of Federal Regulations (CFR) Part 820 (21 CFR Part 820). This document was released by the FDA in 1978 to establish regulatory requirements for the methods, facilities, and controls used to produce medical devices [11]. To meet 21 CFR Part 820 compliance, the FDA requires the establishment of a quality management system (QMS). A QMS functions as a formalized method for documenting processes, procedures, and responsibilities to ensure that medical devices meet specified requirements. Within the QMS, the FDA requires the use of a document control system (DCS) that is compliant with CFR Part 11, the FDA guidelines for electronic record collection [12]. A DCS is a formalized process for the organization, tracking, signature collection, and distribution of manufacturing documentation. While off-the-shelf solutions for a DCS exist, these are often costly and require use of Software as a Service systems which hospitals are hesitant to adopt due to network security concerns. To overcome these challenges, we present our internally developed, cost-effective solution for an electronic DCS for 3D printing at the point-of-care.

In anticipation of FDA regulation of 3D printing and to ensure that our printed parts are of the highest quality, our 3D printing team created an FDA-compliant DCS that provides a cheaper, tailored alternative to traditional solutions. We developed a hybrid system that uses an existing document management program to create an internally managed DCS. Our solution provides a streamlined workflow that can be easily implemented and modified. Our system is also capable of supplying document templates, tracking version history, and collecting electronic signatures, all while automatically generating an FDA-compliant audit trail. The hybrid document control platform can be easily implemented at a low cost by any 3D printing team in a clinical environment, preparing this new sector of healthcare for FDA regulation.

## Methods

### Document control system requirements for 3D printing

Before developing our document control system, we outlined system requirements based on existing guidance. Requirements were developed according to 21 CFR Part 820 and CFR Part 11. We divided requirements into two categories. The first category contains required features. These are features that are necessary for FDA compliance and overall quality assurance. The second category contains additional desired features which are nonessential. Rather, these are features that make the platform easier to use, further streamline the workflow, and expand the system’s applications. These requirements are described below in Table 1.

**Table 1:** Features for our 3D printing DCS.

Required features	Desired features
Document control (versioning, archiving, etc.)	Structure and templates designed for medical device development
Electronic signoffs/approvals with CFR Part 11 compliance	Connectivity between documents (live links to referenced documents)
Ability to store overarching standard operating procedures, work instructions, records, etc. related to program’s infrastructure/policies (Word documents, Excel spreadsheets)	Design history file (DHF) creation
Ability to sort standard operating procedures, work instructions, records, etc. by project (Word documents, Excel spreadsheets)	Complaint management
Creation of FDA-compliant audit documents	Change order management
	Corrective and preventative action (CAPA) subsystem
	Management of employee training
	Consulting services (related to CFR Part 820)
	Risk management capabilities

### Evaluation of existing software applications

After outlining system requirements, we evaluated existing software applications. The applications we explored included Greenlight Guru (Greenlight Guru, IN, USA), Qualio (Qualio, CA, USA), Solidworks PDM (SolidWorks Corporation, MA, USA), Compass (Cognition Corporation, MA, USA), MasterControl (MasterControl, Inc., UT, USA), ETQ (Hexagon AB, Stockholm, Sweden), Arena (PTC, Inc., MA, USA), Qualtrax (Ideagen PLC, Nottingham, United Kingdom), and TrackWise (Sparta Systems, Inc., NJ, USA). Many of these applications have well-designed user interfaces and technical capabilities to meet all required and desired features. Unfortunately, these programs are expensive with costs significantly increasing as the number of dedicated users increases. Additionally, security concerns pose a significant challenge when working with external software platforms. Patient information must be protected, and hospital software security standards must be followed.

Customized internal systems, on the other hand, can be designed to create individualized document control solutions with an internal support team and acceptable security protocols. The downside is that creating a completely custom DCS takes a significant amount of time and can be significantly expensive.

### Creation of a hybrid document control system

Our proposed solution is a hybrid document control system. We have linked two commercially available platforms, Microsoft SharePoint (Microsoft Corporation, WA, USA) and Adobe Sign (Adobe, CA, USA), using a customized workflow which allows for robust document management.

We adapted SharePoint, a Microsoft product for sharing and editing documents, along with Adobe Sign, a Part 11-compliant electronic signature collection service, to create a traceable DCS. The two platforms are connected by Microsoft Power Automate, a programming application that allows for the development of automated workflows between various Microsoft and non-Microsoft platforms. With this automation, a document created in SharePoint can be assigned to reviewers and submitted for review and electronic signatures. The program automatically sends the document to assigned reviewers and collects signatures via Adobe Sign. During the entire process, documents are actively versioned and stored in a *Review Queue* where users can see who has seen, approved, or rejected various documents.

**Fig. 1:**
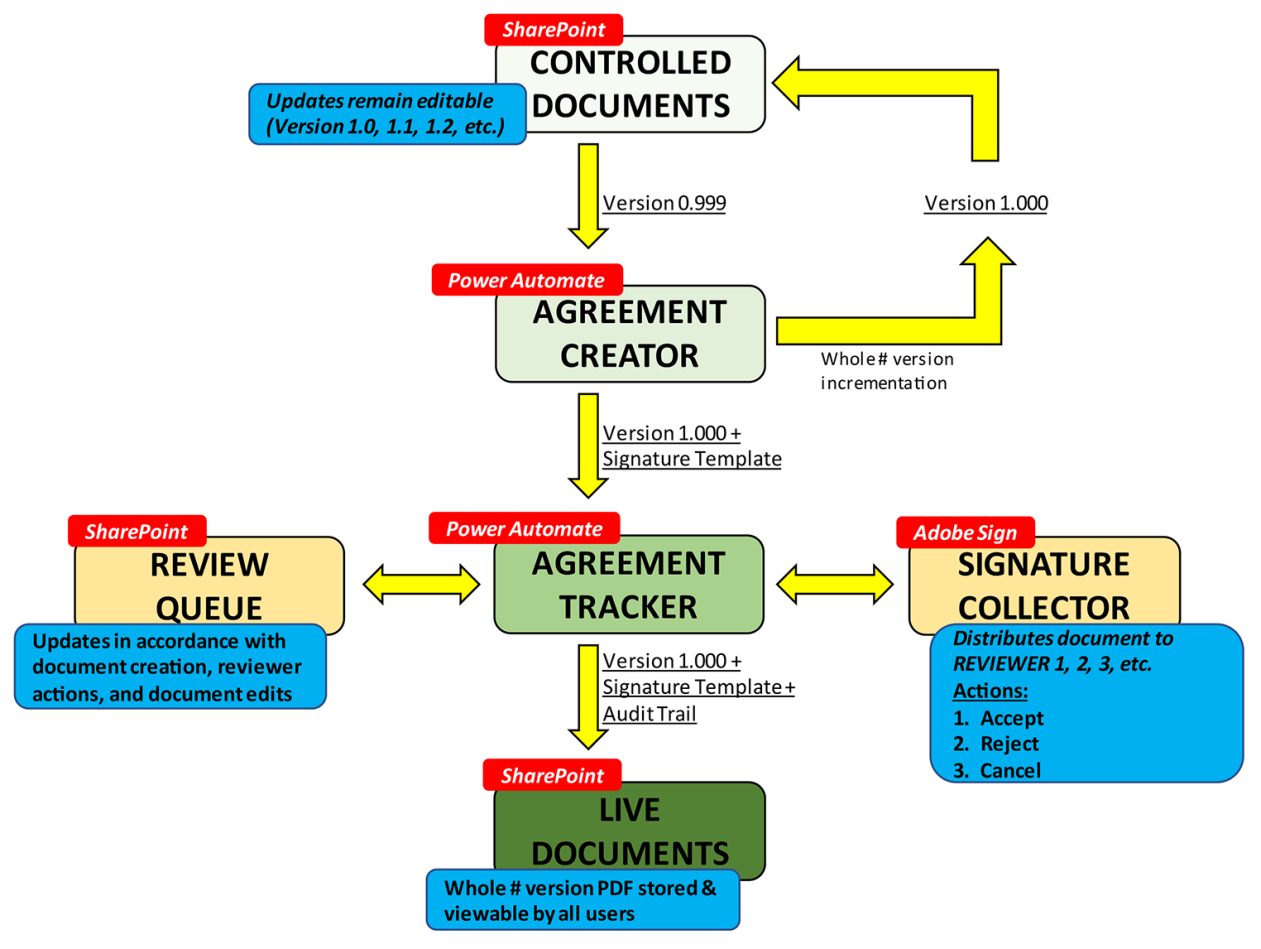
Schematic diagram of our hybrid DCS.

Our document control platform is structured in six sections, demonstrated in Fig. 1. Within Fig. 1, each section is labeled by a red box as either a SharePoint library, Power Automate program, or an Adobe Sign signature collector. The blue boxes summarize the function of a particular section. Yellow arrows represent the movement of a document from section to section.

The first section, *Controlled Documents*, is where a user selects a template for document creation. Within this section, a user can edit the new document, select a hierarchy of reviewers, and send the document for review. After sending the document for review, the *Agreement Creator* is initiated. This section is a Power Automate program that creates an iterated version of the document for review and combines it with an Adobe Sign template. This combination of document and template are referred to as an “Agreement” within Adobe Sign.

The Adobe Sign agreement is then funneled to the *Agreement Tracker*. The *Agreement Tracker* is a separate Power Automate program that tracks the status of an agreement within Adobe Sign’s *Signature Collector* and reflects status changes to users via a SharePoint list called *Review Queue*. The *Signature Collector* is housed within Adobe Sign to collect signatures and create an audit trail. Reviewers can perform three major actions; they can accept, reject, or cancel the agreement. If the reviewer performs any of these three actions, an update is sent to the *Agreement Tracker* section which is further reflected in the *Review Queue*. Rejecting or canceling the agreement automatically sends the document back to the document creator with comments and edits from the reviewer. If an agreement has been reviewed, accepted, and signed by all parties, the *Agreement Tracker* converts the Agreement to a PDF document, attaches the audit trail generated by Adobe Sign, and saves the document to the final section of the software, the *Live Documents* library. Documents in the *Live Documents* library are considered official published records and can be viewed by all users.

## Results

For a DCS to be FDA compliant, it must meet the guidances of CFR Part 11 for electronic signature collection and document protection. Within our system, we use Adobe Sign which is already considered Part 11 compliant as a legally binding signature collection platform. Table 2 outlines all CFR Part 11 subsections and specifies how our system meets each guideline.

**Table 2:** CFR part 11 guidances and how our DCS accounts for each subsection

CFR Part 11 subsection	Subsection guideline	How our system meets guideline
11.10 (a)	Validation of systems	Performance qualification document generated with FDA audit trail
11.10 (b)	Generation of accurate and complete digital and physical copies of records	Documents are stored as Microsoft Word documents and PDF files
11.10 (c)	Protection of records	SharePoint-managed groups and permissions with account passwords
11.10 (d)	Access limitations	SharePoint-managed groups and Microsoft account passwords Individual documents can be further password protected if necessary
11.10 (e)	Creation of time-stamped audit trails	SharePoint tracks all changes to document versions Adobe Sign tracks the review and approval process These features combine to form a complete audit trail
11.10 (f)	Operational system checks	The workflow is managed by Power Automate which is effectively the operational system Every run of the system can be examined at each process step and evaluated for completion and elapsed time
11.10 (g)	Authority checks	Access to the SharePoint library and its documents is managed by a SharePoint administrator who assigns user privileges Adobe Sign requires pre-selected document reviewers to login and defines user privileges in accordance with their assigned roles
11.10 (h)	Device checks	UPMC login is required to access the local network on which SharePoint is hosted Users must sign into their Microsoft account to access the SharePoint document library
11.10 (i)	Proof of qualifications	Employee training documents are available for and reviewed by all system users
11.10 (j)	Establishment of policies that hold signers responsible under their electronic signatures	E-signature disclaimer on the coversheet seen in Adobe Sign at the time of signature
11.10 (k1)	Control over distribution, access, and use	All process documents are stored in SharePoint under carefully assigned and controlled user permissions
11.10 (k2)	Revision and change control procedure and their audit trail	Documentation pertaining to this document control process will be kept under the same protocols it enforces Document libraries include SharePoint versioning and Adobe Sign audit trails
11.50 (a)	Signed records must contain the signer’s printed name, date, and time of signature, and the meaning of the signature	All fields are present on the Adobe Sign Template All informatization, minus the signature, is automatically generated at the time of signature
11.50 (b)	All information required in 11.50 (a) will be available to the reader in both digital and physical format	Signed document is exported to the SharePoint *Live Documents* library as a PDF file, which has the signatures and associated information on the coversheet
11.70	Signature/record linking	Managed and controlled by Adobe Sign which is a Part 11 compliant e-signature application

Section 11.30 of Part 11 is not considered because we operate within a closed system, internal to UPMC. All other sections were carefully examined when creating our platform.

## Discussion

Our hybrid DCS provides an affordable, customizable option for point-of-care 3D printing groups. Using a hybrid approach, we leverage the advantages of both existing software platforms and customized workflows. Microsoft SharePoint is a sophisticated interface for document sharing across various institutions. The program is compatible with all Microsoft Office products including Word and Excel, two widely used document creation programs. These products are used across many institutions, making our system a viable option to implement. With the help of Power Automate, we were able to design a workflow that allows us to version documents, archive edits, assign reviewers, and collect signatures in a format that is FDA compliant. Broadly speaking, any 3D printing program with access to a Microsoft Enterprise account can implement our document control solution.

Throughout this process, access to documents is controlled on a document library level. A core feature of SharePoint is the ability to assign read, write, and edit privileges to specific users or user groups. Both the *Controlled Documents* and *Live Document* libraries have restricted access to avoid the intentional or accidental creation of “uncontrolled” documents. In summary, SharePoint is used to store and edit documents; it contains the necessary components of Part 11 except for the regulations surrounding electronic signatures. The regulations surrounding electronic signatures, in turn, are met by Adobe Sign. The resulting DCS can store documents and collect signatures all within a transparent, traceable format.

Future goals for improvement are to allow system users to actively change and manage orders within the review workflow. We would also like to link documents with electronic health records when applicable. Other improvements will include risk management workflows and the automatic creation of design history files to further track 3D printing activities.

## Conclusion

Overall, regardless of external regulation, our 3D printing team strives to provide high-quality additive manufacturing support to our healthcare community. To meet the highest of standards, a detailed DCS is necessary to define protocols, track information, and process prints at a high volume. Our hybrid solution for document control provides an affordable system for users to sort, manage, store, edit, and sign documents. We hope our system can serve as a framework for other 3D printing programs to prepare for future FDA regulations, improve the efficiency of 3D printing at the point-of-care, and enhance the quality of work produced by their respective program.

## Data Availability

Power Automate programming is available from the corresponding author on reasonable request.

## List of Abbreviations

CAPA: Corrective and Preventative Action.
CFR: Code of Federal Regulations.
DCS: Document Control System.
DHF: Design History File.
FDA: United States Food and Drug Administration.
QMS: Quality Management System.
